# Supplementary material for: A Neuroprotective Peptide Modulates Retinal cAMP Response Element-Binding Protein (CREB), Synapsin I (SYN1), and Growth-Associated Protein 43 (GAP43) in Rats with Silicone Oil-Induced Ocular Hypertension
Source: Biomolecules. 2025 Feb 3;15(2):219. doi: 10.3390/biom15020219 (PMC11852426; doi:10.3390/biom15020219)
Supplement: Supplementary file 1 [file biomolecules-15-00219-s001.zip › biomolecules-3277128-supplementary.pdf]

biomolecules

MDPI

Article

A Neuroprotective Peptide Modulates Retinal cAMP Response Element-Binding Protein (CREB), Synapsin I (SYN1), and Growth-Associated Protein 43 (GAP43) in Rats with Silicone Oil-Induced Ocular Hypertension

Gretchen A. Johnson [1,2], Raghu R. Krishnamoorthy [1,3], Ram H. Nagaraj [4] and Dorota L. Stankowska [1,2,*]

Supplementary Materials

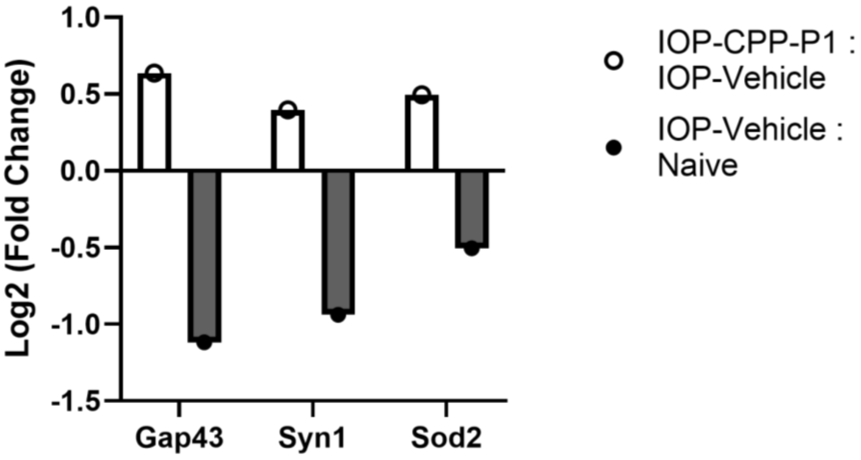

Figure S1. RNA Expression from RGCs.

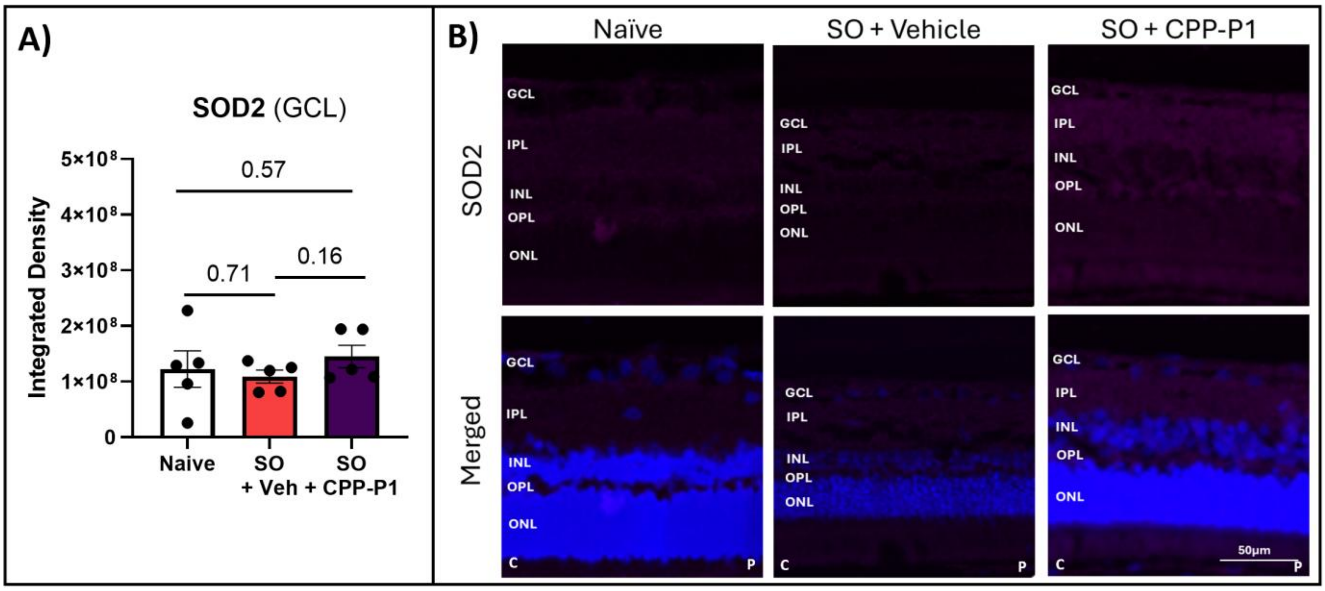

Figure S2. Mitochondrial SOD2. (A) Ganglion Cell Layer fluorescence. (B) Representative images.

 https://doi.org/10.3390/biom15020219
